# Supplementary material for: Sugar consumption in schoolchildren from southern Spain and influence on the prevalence of obesity
Source: PLoS One. 2020 Nov 20;15(11):e0242602. doi: 10.1371/journal.pone.0242602 (PMC7678956; doi:10.1371/journal.pone.0242602)
Supplement: Attachment — Submitted filename: Review Comments.docx [file pone.0242602.s001.docx]

Dear Editor,

Referee's evaluation form

MS ID: PONE-D-20-10138

Title: Sugar consumption in school children from southern Spain and influence on the prevalence of obesity

Comments - further recommendations

I hope these comments are helpful.

This submitted manuscript evaluated the impact of sugar consumption on prevalence of obesity in school children of Granada and Malaga, Spain. The subject of the paper is interesting that provides new data and the scope covered by the manuscript is worth to be published in PLOS One. The abstract and keywords are fine. However, the article may be reconsidered for publication after the authors make some minor revisions.

Comments to the Authors:

1. Line 123: Methods section: Out of the initial sample of 700 individuals 42 subjects were excluded. That leaves the total sample size of 658. However, author reported that study conducted on 657 schoolchildren. Also, in the tables 3 and 4, the cumulative sample size is 631 and 630, respectively. Were there any drop-out from the subjects during the study period? If there were any drop-outs, I would recommend to mention this either in method or discussion section.
