# Supplementary material for: Sugar consumption in schoolchildren from southern Spain and influence on the prevalence of obesity
Source: PLoS One. 2020 Nov 20;15(11):e0242602. doi: 10.1371/journal.pone.0242602 (PMC7678956; doi:10.1371/journal.pone.0242602)
Supplement: Attachment — Submitted filename: plosoneNEWREVISED1820.docx [file pone.0242602.s002.docx]

**Comments to the Author**

1. Is the manuscript technically sound, and do the data support the conclusions?

The manuscript must describe a technically sound piece of scientific research with data that supports the conclusions. Experiments must have been conducted rigorously, with appropriate controls, replication, and sample sizes. The conclusions must be drawn appropriately based on the data presented.

Reviewer #1: Yes

Reviewer #2: Partly

Reviewer #3: Partly

2. Has the statistical analysis been performed appropriately and rigorously?

Reviewer #1: Yes

Reviewer #2: Yes

Reviewer #3: No

3. Have the authors made all data underlying the findings in their manuscript fully available?

The PLOS Data policy requires authors to make all data underlying the findings described in their manuscript fully available without restriction, with rare exception (please refer to the Data Availability Statement in the manuscript PDF file). The data should be provided as part of the manuscript or its supporting information, or deposited to a public repository. For example, in addition to summary statistics, the data points behind means, medians and variance measures should be available. If there are restrictions on publicly sharing data—e.g. participant privacy or use of data from a third party—those must be specified.

Reviewer #1: Yes

Reviewer #2: Yes

Reviewer #3: Yes

4. Is the manuscript presented in an intelligible fashion and written in standard English?

PLOS ONE does not copyedit accepted manuscripts, so the language in submitted articles must be clear, correct, and unambiguous. Any typographical or grammatical errors should be corrected at revision, so please note any specific errors here.

Reviewer #1: Yes

Reviewer #2: Yes

Reviewer #3: Yes

5. Review Comments to the Author

Please use the space provided to explain your answers to the questions above. You may also include additional comments for the author, including concerns about dual publication, research ethics, or publication ethics. (Please upload your review as an attachment if it exceeds 20,000 characters)

**Reviewer #1:** Comments to the Authors:

1. Line 123: Methods section: Out of the initial sample of 700 individuals 42 subjects were excluded.

That leaves the total sample size of 658. However, author reported that study conducted on 657 schoolchildren. Also, in the tables 3 and 4, the cumulative sample size is 631 and 630, respectively. Were there any drop-out from the subjects during the study period? If there were any drop-outs, I would recommend to mention this either in method or discussion section.

**Response:** The drop-outs detected by the reviewer were largely due to missing data on sex or height.

Reviewer #2: The study design can be followed. Critical point: What is new? The results simply confirm some previous studies. Please, make a statement on that point.

**Response:** A simple open review of the literature was conducted in Pubmed, with *sugar intake* and *schoolchildren* as search terms. It yielded a total of 13 scientific articles from the past 10 years. The WHO report on “*Guideline: Sugars intake for adults and children”* was published in 2015, and our article offers a response to the constant stream of misleading reports with little or no scientific basis on the consumption of sugar in its different forms. Our study, which offers information on the intake of sugars by Spanish schoolchildren and its relationship with their BMI, contributes to previously published data on this important issue, including the following articles:

ANNEX I

Cabrera Escobar MA, Veerman JL, Tollman SM, Bertram MY, Hofman KJ.Evidence that a tax on sugar sweetened beverages reduces the obesity rate: a meta-analysis. BMC Public Health. 2013 Nov 13;13:1072. doi: 10.1186/1471-2458-13-1072

Dudley DA, Cotton WG, Peralta LR Teaching approaches and strategies that promote healthy eating in primary school children: a systematic review and meta-analysis. . Int J Behav Nutr Phys Act. 2015 Feb 25;12:28. doi: 10.1186/s12966-015-0182-8.

Gates A, Skinner K, Gates M. The diets of school-aged Aboriginal youths in Canada: a systematic review of the literature. J Hum Nutr Diet. 2015 Jun;28(3):246-61. doi: 10.1111/jhn.12246. Epub 2014 May 22

Gibson S Sugar-sweetened soft drinks and obesity: a systematic review of the evidence from observational studies and interventions. Nutr Res Rev. 2008 Dec;21(2):134-47. doi: 10.1017/S0954422408110976

Gupta A, Kapil U, Singh G.Consumption of junk foods by school-aged children in rural Himachal Pradesh, India. Indian J Public Health. 2018 Jan-Mar;62(1):65-67. doi: 10.4103/ijph.IJPH_343_16

Huang JY, Qi SJ. Childhood obesity and food intake World J Pediatr. 2015 May;11(2):101-7. doi: 10.1007/s12519-015-0018-2. Epub 2015 Apr 30

López del Val T, Estivariz CF, Martínez de Icaya P, Jaunsolo MA, del Olmo D, Vázquez Martínez C.Consumption of sweets and snacks by a population of school children in the Autonomous Community of Madrid. The CAENPE Group]. Med Clin (Barc). 1997 Jun 14;109(3):88-91

Nasreddine LM, Kassis AN, Ayoub JJ, Naja FA, Hwalla NC Nutritional status and dietary intakes of children amid the nutrition transition: the case of the Eastern Mediterranean Region. Nutr Res. 2018 Sep;57:12-27. doi: 10.1016/j.nutres.2018.04.016. Epub 2018 Apr 26

Papanikolaou Y, Jones JM, Fulgoni VL Several grain dietary patterns are associated with better diet quality and improved shortfall nutrient intakes in US children and adolescents: a study focusing on the 2015-2020 Dietary Guidelines for Americans. Nutr J. 2017 Feb 20;16(1):13. doi: 10.1186/s12937-017-0230-0.

Rouhani MH, Salehi-Abargouei A, Azadbakht L. Effect of glycemic index and glycemic load on energy intake in children.Nutrition. 2013 Sep;29(9):1100-5. doi: 10.1016/j.nut.2013.02.004. Epub 2013 Jun 6

Sahoo K, Sahoo B, Choudhury AK, Sofi NY, Kumar R, Bhadoria AS. Childhood obesity: causes and consequences. J Family Med Prim Care. 2015 Apr-Jun;4(2):187-92. doi: 10.4103/2249-4863.154628

Shamah-Levy T, Cuevas-Nasu L, Gómez-Acosta LM, Morales-Ruan MDC, Méndez-Gómez Humarán I, Robles-Villaseñor MN, Hernández-Ávila M. Effects of SaludArte program in feeding and nutrition components in school children in Mexico City]. Salud Publica Mex. 2017 Nov-Dec;59(6):621-629. doi: 10.21149/8116

Tabatabaei-Malazy O, Nikfar S, Larijani B, Abdollahi M Influence of ascorbic acid supplementation on type 2 diabetes mellitus in observational and randomized controlled trials; a systematic review with meta-analysis. J Pharm Pharm Sci. 2014;17(4):554-82. doi: 10.18433/j3zg6r

Introduction. Several wordings should be corrected.

Lines 72f: From the physiological point of view, the cause of weight gain in otherwise healthy people is always (!!) in all ages an imbalance between energy intake and energy consumption;

**Response:** The adverb “always” has been changed to “usually”.

line 77: What is meant with "poor diet"? Please define.

**Response:** A poor diet is now exemplified in this context as an excessive energy intake *“(e.g., excessive energy consumption)”*

lines 100f: It should be correctly mentioned that the consumption of energy(!)-containing beverages increase the risk to develop an energy imbalance - this is not only related to carbohydrates present in beverages.

**Response:** We have added the following qualification in the revised text:

*It has been reported that children who consume more sugar-sweetened beverages are more likely to be overweight or obese, although this would be attributable not only to the carbohydrates in these drinks but also to the lipids and proteins they contain.*

Methods. Recruitment: All necessary criteria with respect to sample point selection, number of participants planned, exclusion criteria etc. must be given - is the study representative for Spain?

**Response:** This population derives from a health research project of the Spanish Ministry of Health FEDER-ISCIII PI14/01040. Written informed consent was obtained from parents/guardians of all participants in the study, which was approved by the research ethics committee of the of the Andalusian Public Health Service. Two age groups were defined in the total sample of 1,000 schoolchildren: a subgroup of children aged 7-8 years and another subgroup aged 9-10 years. The sample comprised schoolchildren from the provinces of Granada and Malaga. Sample size: For an expected obesity prevalence of 10 % and precision of 2 %, with a 95% confidence interval, the sample size would be 540 (P: 0.10; i: 0.02; α: 0.05). After correction for a finite population: nα = 540/ (1+[540/2940]) = 456, the minimum sample size was 456 individuals. We only excluded children whose parents/guardians refused participation.

Regression analysis: Why only carbohydrates taken into account? Why not fat intake? According to table 1, fat intake showed a broader variation than the sum of carbohydrate intake!

**Response:** Besides carbohydrates, we considered the total energy/day, including carbohydrates, lipids and proteins.

Discussion. Strengths/weaknesses of the study design must be discussed, e.g., the compliance of data collection and the recruitment (see above). Most of the first 2 paragraphs are a repetition of results - can be shortened.

**Response:** The first two paragraphs have been shortened, as requested, and we now discuss strengths/weaknesses at the end of the Discussion, as follows:

*“The present results are confined to schoolchildren aged 7 to 10 years and do not consider nutritional and life habits that may be acquired in adolescence. A further limitation is that only the intake of sugars and total energy was studied, and the contribution of other energy nutrients (e.g., proteins and fats) was not taken into account. “*

What about studies in other European countries like Germany? Eg, KOPS? Line 211f:

**Response:** As shown by the list of publications cited above, few published data are available. However, we have now included the following studies from KOPS, despite being published more than a decade ago:

Danielzik S, Pust S, Müller MJ. School-based interventions to prevent overweight and obesity in prepubertal children: process and 4-years outcome evaluation of the Kiel Obesity Prevention Study (KOPS). Acta Paediatr. 2007 Apr;96(454):19-25. doi: 10.1111/j.1651-2227.2007.00165.x. PMID: 17313410.

Landsberg B, Plachta-Danielzik S, Much D, Johannsen M, Lange D, Müller MJ. Associations between active commuting to school, fat mass and lifestyle factors in adolescents: the Kiel Obesity Prevention Study (KOPS). Eur J Clin Nutr. 2008 Jun;62(6):739-47. doi: 10.1038/sj.ejcn.1602781. Epub 2007 May 16. PMID: 17522617.

What is meant with "unhealthy" food?

We have now added the following description in the revised paper: *The most common unhealthy foods include highly-processed items (e.g., fast foods and snacks), which tend to be low in nutrients (vitamins, minerals and antioxidants) and high in empty calories due to the content of refined flours, sodium and sugar.*

Criteria? And a "poorly balanced diet"? Please, explain.

**Response:** For this population in the Mediterranean area, a poorly balanced diet was defined by food habits that are not compatible with the Mediterranean Diet. The KIDMED index was used as a variable in our study to determine adherence/non-adherence to this well-documented dietary model.

Conclusion. Since only carbohydrate intake was taken into account, the conclusion is not valid.

**Response:** We have modified the conclusion accordingly. It now reads:

*In conclusion, t**he reduction of sugar intake, alongside the limitation of total daily intake from energy nutrients such as fats and proteins, is essential to combat the alarming prevalence of obesity among children. Well-designed programs are required that involve parents and schools. Further studies are warranted to verify these findings and examine their implication for public health policies*

Reviewer #3: The authors present an interesting dataset about sugar consumption and the prevalence of obesity in schoolchildren from southern Spain. However, the result is poorly structured and suffers from inappropriate data presentation and analysis as detailed below.

1. The cut-offs for normal weight, overweight, and obesity were not given.

**Response:** We followed the cutoffs published by Cole et al, (2012) in their table (by sex and age)

| Age (years) | Body mass index 25 kg/m2 |  | Body mass index 30 kg/m2 |  |
| --- | --- | --- | --- | --- |
|  | Males | Females | males | females |
| 7 | 17.92 | 17.75 | 20.63 | 20.51 |
| 8 | 18.44 | 18.35 | 21.60 | 21.57 |
| 9 | 19.10 | 19.07 | 22.77 | 22.81 |
| 10 | 19.84 | 19.86 | 24.0 | 24.11 |
|  |  |  |  |  |

2. The results were barely interpreted:

a. Per Table 1, I would suggest presenting the data by weight status, i.e. underweight, normal weight, overweight, and obesity. Is any participant underweight?

**Response** :There were no underweight children in the study population

b. How did the authors conclude “industrial milkshakes were the predominant source of total carbohydrates representing 50.9%” (Line 166-168) from Table 2?

**Response.** We used stepwise regression analysis, following Willet, (1998), to analyze the correlation between the components of foods and the intake of each.

c. What are the definitions of total sugars, free sugars, and added sugars? Are they overlapped? What were their effects on body weight, respectively?

**Response:** Indeed, there are various complementary definitions of these terms, as follows:

*The European Food Safety Authority (EFSA) defines sugars as ‘‘total sugars,’’ including both indigenous sugars naturally present in foods (ie, ‘‘naturally occurring sugars’’) such as fruit, vegetables, cereals, and lactose in milk products, and added sugars. The term ‘‘added sugars’’ refers to sucrose, fructose, glucose, starch hydrolysates (glucose syrup, high-fructose syrup, isoglucose), and other isolated sugar preparations used as such, or added during food preparation and manufacturing*

EFSA. Review of labelling reference intake values. Scientific Opinion of the Panel on Dietetic Products, nutrition and allergies on a request from the Commission related to the review of labelling reference intake values for selected nutritional elements. EFSA J 2009;1008:1–14.

*Free sugars include monosaccharides and disaccharides added to foods and beverages by the manufacturer, cook or consumer, and sugars naturally present in honey, syrups, fruit juices and fruit juice concentrates. For countries with a low intake of free sugars, levels should not be increased. Higher intakes of free sugars threaten the nutrient quality of diets by providing significant energy without specific nutrients (3).* Guideline: sugars intake for adults and children. World Health Organization. 2015 Guideline: Sugars Intake for Adults and ISBN 978 92 4 154902 8 (NLM classification: QU 145.7)

© World Health Organization, 2015 World Health Organization.

d. Did the authors perform analysis of covariance when investigating the association of sugar

consumption and obesity?

**Response:** This analysis was performed but did not yield additional results of interest

3. Some sentences are confusing:

*Line 64-66: “The likelihood of normal weight was significantly greater with lower energy expenditure in sedentary activities (OR=3.03), higher energy expenditure in sports activities (OR=1.72), and total activity/day measured as METs (OR=8.31).”*

**Response:** *This now reads:*

*“The likelihood of normal weight was significantly greater with lower energy expenditure in sedentary activities (OR=3.03), higher energy expenditure in sports activities (OR=1.72), and higher total activity/day measured as METs (OR=8.31).”*

Similarly, line 177-178: *“Normal weight was found to be associated with physical activity, as measured by METs or total energy expenditure/day, and with energy intake.”*

**Response:** This line has been eliminated in the revised version (after the requested reduction in first two paragraphs).
