# Supplementary material for: Sugar consumption in schoolchildren from southern Spain and influence on the prevalence of obesity
Source: PLoS One. 2020 Nov 20;15(11):e0242602. doi: 10.1371/journal.pone.0242602 (PMC7678956; doi:10.1371/journal.pone.0242602)
Supplement: Attachment — Submitted filename: Response to Reviewers.docx [file pone.0242602.s003.docx]

**Comments to the Author**

1. If the authors have adequately addressed your comments raised in a previous round of review and you feel that this manuscript is now acceptable for publication, you may indicate that here to bypass the “Comments to the Author” section, enter your conflict of interest statement in the “Confidential to Editor” section, and submit your "Accept" recommendation.

Reviewer #1: All comments have been addressed

Reviewer #2: (No Response)

Reviewer #3: (No Response)

2. Is the manuscript technically sound, and do the data support the conclusions?

The manuscript must describe a technically sound piece of scientific research with data that supports the conclusions. Experiments must have been conducted rigorously, with appropriate controls, replication, and sample sizes. The conclusions must be drawn appropriately based on the data presented.

Reviewer #1: Yes

Reviewer #2: Yes

Reviewer #3: Yes

3. Has the statistical analysis been performed appropriately and rigorously?

Reviewer #1: Yes

Reviewer #2: Yes

Reviewer #3: Yes

4. Have the authors made all data underlying the findings in their manuscript fully available?

The PLOS Data policy requires authors to make all data underlying the findings described in their manuscript fully available without restriction, with rare exception (please refer to the Data Availability Statement in the manuscript PDF file). The data should be provided as part of the manuscript or its supporting information, or deposited to a public repository. For example, in addition to summary statistics, the data points behind means, medians and variance measures should be available. If there are restrictions on publicly sharing data—e.g. participant privacy or use of data from a third party—those must be specified.

Reviewer #1: Yes

Reviewer #2: Yes

Reviewer #3: Yes

5. Is the manuscript presented in an intelligible fashion and written in standard English?

PLOS ONE does not copyedit accepted manuscripts, so the language in submitted articles must be clear, correct, and unambiguous. Any typographical or grammatical errors should be corrected at revision, so please note any specific errors here.

Reviewer #1: Yes

Reviewer #2: Yes

Reviewer #3: Yes

6. Review Comments to the Author

Please use the space provided to explain your answers to the questions above. You may also include additional comments for the author, including concerns about dual publication, research ethics, or publication ethics. (Please upload your review as an attachment if it exceeds 20,000 characters)

Reviewer #1: This submitted manuscript evaluated the impact of sugar consumption on prevalence of obesity in school children of Granada and Malaga, Spain. The subject of the paper is interesting that provides new data and the scope covered by the manuscript is worth to be published in PLOS One. The abstract and keywords are fine. The manuscript is well revised and the authors have made appropriate changes as suggested, and thus, I recommend to publish the manuscript in its current form.

**Response:** The authors are grateful for the positive evaluation of Reviewer 1.

Reviewer #2: Obviously, the authors generally answered the questions in their response commentaries adequatly; however, the text was not always changed accordingly. Some examples:

1. Recruitment: The text formulated as response to the reviewers` question should be integrated completely in the Methods section!

2. Line 102: The authors should simply write "energy-containing beverages" and add in brackets (optional) "carbohydrates, fat and protein".

3. Line 77: If a "hyperenergetic diet" is meant, then the authors should use this term! A "poor diet" is not only a question of energy content.

4. Line 215f: The term "unhealthy food" is simply not correct in this context. The study only evaluate "energy intake" with respect to body weight and does not focus on e.g. micronutrients. This sentence must be reworded.

**Response:** On point 1, the authors have completely included the text in the Methods section (lines 122-134). In relation to point 2, the authors have changed "sugar-sweetened beverages" to "energy-containing beverages”. Point 3: The authors have changed “poor diet” to “hyperenergetic diet”. Point 4: The authors have changed “unhealthy food” to “sugary and processed food” (lines 219-220 and 222).

Reviewer #3: The authors did a good job addressing all my questions and concerns except one.

Regarding Table 2 (Line 171-172), the authors interpreted the data incorrectly. R squared (60.9% rather than 50.9%, 74.7-82%) in Stepwise regression analysis doesn't equal to % of the contribution. I would suggest authors add a column in Table 2 as % of contribution and correct the data accordingly in Line 171-172.

**Response:** The authors appreciate the good feedback. The proposed changes to lines 171-172 have been made (lines 177-178 revised manuscript). Also, the authors have updated Table 2 by including the % contribution column.

7. PLOS authors have the option to publish the peer review history of their article (what does this mean?). If published, this will include your full peer review and any attached files.

If you choose “no”, your identity will remain anonymous but your review may still be made public.

Do you want your identity to be public for this peer review? For information about this choice, including consent withdrawal, please see our Privacy Policy.

Reviewer #1: No

Reviewer #2: No

Reviewer #3: No
